# Growing Apart: A Longitudinal Assessment of the Relation Between Post-traumatic Growth and Loneliness Among Combat Veterans

**DOI:** 10.3389/fpsyg.2018.00893

**Published:** 2018-06-07

**Authors:** Jacob Y. Stein, Yafit Levin, Rahel Bachem, Zahava Solomon

**Affiliations:** ^1^I-CORE Research Center for Mass Trauma, Tel Aviv University, Tel Aviv, Israel; ^2^Bob Shapell School of Social Work, Tel Aviv University, Tel Aviv, Israel

**Keywords:** loneliness, post-traumatic growth, PTG, war trauma, veterans, experiential loneliness

## Abstract

The aftermath of war-related trauma may entail psychological devastation and is typically accompanied by various deleterious phenomena. These include, but are not limited to, high rates of loneliness. However, trauma may also result in positive outcomes such as personal, spiritual, and relational prosperity, which are typically considered under the conceptual framework of post-traumatic growth (PTG). PTG may theoretically contribute to either loneliness amelioration (e.g., via increasing one’s appreciation of close relationships) or exacerbation (e.g., by increasing one’s sense of undergoing experiences that others do not share). Loneliness, on the other hand, may potentially hinder PTG by fostering negative social cognitions and behaviors, or otherwise lead to personal growth. The relations between the two phenomena, however, have yet to be investigated. Filling this gap, the current study examined the aforementioned potentialities by utilizing an autoregressive cross-lagged modeling strategy (ARCL) with a cohort of 260 Israeli combat veterans assessed 30, 35, and 42 years after their participation in the 1973 Yom Kippur War. Results indicated that higher rates of PTG were consistently related to higher rates of loneliness both cross-sectionally and longitudinally. Loneliness, however, did not longitudinally predict PTG rates. It is suggested that these findings may be understood in light of the observation that veterans’ loneliness is primarily related to the experience of being experientially out of sync with people who have not endured war experiences. It is suggested that this *experiential loneliness* may include not only the negative but also the positive ramifications of undergoing such traumas (i.e., PTG). We, therefore, argue that while PTG may include authentic positive transformations it may also lead to more negative ramifications, and these should be identified and addressed by researchers and clinicians alike. Thus, as study limitations are acknowledged, clinical implications, and future research directions are suggested.

*I only grow apart from people who don’t grow*.

- Jaime Primak Sullivan

## Introduction

The stressors of war (Nash, 2007) and war captivity (Hunter, 1993) are vast and multifaceted, and many of them are of an interpersonal nature (e.g., humiliations, psychological abuse, punitive torture; Stein et al., 2015). Thus, for the most part, the study of war-induced trauma has been, and to a great extent still is, focused on the negative ramifications of such experiences. The detrimental aftermaths of war and captivity have been the focus of countless studies, ranging across domains such as post-traumatic stress disorder (PTSD), premature mortality, suicidality, substance abuse, and various ailments and maladies (e.g., Neria et al., 2000; Dikel et al., 2005; Rintamaki et al., 2009; Solomon et al., 2014; Bryan et al., 2015; Fulton et al., 2015; Russell et al., 2015; Lan et al., 2016). Adding to this deleterious aftermath, evidence indicates that veterans may endure a long-lasting sense of loneliness (e.g., Kuwert et al., 2014; Solomon et al., 2015; Stein and Tuval-Mashiach, 2015a).

Given the predominantly negative focus of trauma-related research, one cannot over-emphasize the significance of the change in attention that the turn of the 21^st^ century has brought with it. Pioneered by Martin Seligman, psychologists have argued that psychological investigations should strive to reveal and underscore the positive rather than only the negative aspects of human development (e.g., Seligman and Csikszentmihalyi, 2000; Seligman et al., 2005). This movement has given rise to the now well-established field of *positive psychology*, and more recently the bourgeoning field of a *positive psychiatry* (Jeste et al., 2015). Positive perspectives have been applied differently in various fields of inquiry. Applied to domains of adversity, the field of addiction for instance, has seen the emergence of the concept of *recovery capital* (Granfield and Cloud, 1999; Hennessy, 2017), which highlights the resources that one can harness in an effort to overcome alcohol and drug dependency. Similarly, the fields of trauma and stress management have seen the emergence of resiliency research (e.g., Bonanno, 2005; Bonanno et al., 2011). Concomitantly, theories that are predicated on individuals’ capacity to utilize and preserve personal resources to mitigate the tolls of adversity have gained currency in the field (Hobfoll, 2002). Such theories include, for instance, conservation of resources (COR) theory (Hobfoll, 1989; Hobfoll et al., 2016) the theory of hardiness (e.g., Kobasa, 1979; Eschleman et al., 2010), and more recently a physiological approach to resilience (Horn et al., 2016). The common factor in all such conceptual frameworks is that external (e.g., social support, socioeconomic status) and internal (e.g., personality traits, physiological systems) properties are considered and evaluated as potential coping resources that may ease the detrimental ramifications of adversity. While almost anything may function as a resource, as a definitional note it is argued “that resources can hold value to the extent that they are perceived to help one achieve his or her goals” (Halbesleben et al., 2014, p. 1340). Identifying such resources and recovery capital has consequently become a top priority objective for research and intervention.

An additional positive development in the traumatology field has been the focus on growth (Joseph and Linley, 2008a). To a large extent, this development has been facilitated by the inception of the post-traumatic growth construct (PTG; Tedeschi and Calhoun, 1995, 1996). PTG denotes positive psychological transformations experienced by survivors of highly stressful and traumatic life events. Though the introduction of the PTG construct has been the source of extensive research (Tedeschi and Calhoun, 2004), the specifics are somewhat contested. For instance, Joseph and Linley (2005, p. 263) assert that “growth consists of positive changes in how people value their relationships with other people, in their self-perceptions, and in their life philosophy.” More specifically, the PTG construct, as initially conceptualized by Tedeschi and Calhoun (1996), encapsulates five domains of positive change: (a) enhanced interpersonal relationships, (b) the realization of possibilities in one’s life that were formerly unrecognized, (c) perceiving oneself as stronger and more potent, (d) an increased appreciation of life, and (e) spiritual growth. Invariably, however, PTG is understood as a negotiation of meaning in the aftermath of adversity, contemplating the positive meanings alongside the negative and consciously or unconsciously determining the personal significance of both (Park, 2010).

The guiding notion of the PTG construct (Tedeschi and Calhoun, 2004) is that, much like in the case of traumatization (Janoff-Bulman, 1992), growth is the result of a reevaluation of the assumptive world (Janoff-Bulman, 2006). However, while traumatization unfolds due to core beliefs about oneself and the world being shattered or challenged by the seismic impact of the trauma, in PTG the individual reacts to this “psychic earthquake” by rebuilding an assumptive world that is fortified by the trauma and may assist survivors in their efforts to withstand future adversity. In this sense, PTSD and PTG are both products of the meaning-making process following trauma (Park and Ai, 2006; Park, 2010; Larner and Blow, 2011).

Such growth phenomena have been widely documented amongst former prisoners of war (ex-POWs) and combat veterans (e.g., Erbes et al., 2005; Maguen et al., 2006; Feder et al., 2008; Pietrzak et al., 2010; Larner and Blow, 2011; Zerach et al., 2013; Tsai et al., 2015, 2016b; Morgan et al., 2017). Indeed, as one literature review demonstrates (Schok et al., 2008), from a meaning-making perspective the aftermath of war as reported by veterans includes more positive than negative effects. Moreover, findings suggest that growth among veterans may be the product of several intrapersonal resources such as optimism, sense of control, and self-esteem (Schok et al., 2010).

As both positive and negative ramifications of war and captivity have become well-established, though by all means not exhausted; the time is ripe to inquire as to the positive and negative *outcomes of these outcomes*. Along these lines, researchers have begun addressing the question whether there are positive as well as negative ramifications to finding benefits in the aftermath of adversity (e.g., Helgeson et al., 2006). Thus, positioning PTG within a context of resources and recovery capital, it has been suggested that, on the one hand, PTG should be conceptualized and investigated as an outcome, which is the most common course of research in this domain; but, on the other hand, PTG should also be examined as a coping strategy (Zoellner and Maercker, 2006). Arguably, when PTG is treated as a coping strategy, empirical evidence may support one of two possibilities: either PTG is adaptive, indicating that it is a resource and protective factor, or it is maladaptive, suggesting that it is a risk factor. Studies reviewed by Zoellner and Maercker (2006) inconsistently support both potentialities.

On the positive side, PTG has been found to reduce suicidal ideation in the aftermath of traumas such as earthquakes (Yu et al., 2010) and combat (Bush et al., 2011). Moreover, it has been found that PTG may predict better functioning among veterans struggling with PTSD (Tsai et al., 2015), as well as contribute to their well-being (Karlsen et al., 2006). Similarly, findings in a recent 2-year longitudinal study among veterans have indicated that the personal strength dimension of PTG predicted reduced severity and incidence of PTSD symptomatology (Tsai et al., 2016a). PTG has also been found to predict better treatment outcomes for PTSD patients (Hagenaars and van Minnen, 2010). Furthermore, the accumulation of empirical research in this domain suggests that the construal of positive meaning from war and peacekeeping experiences, particularly when related to combat exposure or high perceptions of threat, may be associated with better psychological adjustment (Schok et al., 2008). Concomitantly, fostering PTG has become a goal sought in the ongoing effort to cultivate resiliency in combat veterans (Tedeschi, 2011; Tedeschi and McNally, 2011), typically viewing PTG as a recovery capital or resource.

In contrast, it was found that PTG in the context of terrorism, for instance, may be associated with several unfortunate consequences, such as the promotion of violence and ethnocentrism (Hobfoll et al., 2007). In the domestic domain, recent research indicates that higher rates of PTG may hinder dyadic adjustment in veteran couples (Lahav et al., 2017). In the more intrapersonal domain findings suggest that PTG may be associated with more negative world assumptions and greater levels of dissociation among veterans (Lahav et al., 2016). Moreover, PTG has been repeatedly associated with more distress and PTSD, though not necessarily in a linear fashion (e.g., Shakespeare-Finch and Lurie-Beck, 2014). Such findings have led some researchers (Westphal and Bonanno, 2007) to draw the line differentiating PTG from resilience, while others (e.g., Hobfoll et al., 2007; Sumalla et al., 2009) suggest that PTG may represent a “positive illusion,” to borrow the term from Taylor and Armor (1996). Similarly, suggesting a comprehensive model of PTG, Zoellener and Maercker have argued that PTG represents a Janus-faced phenomenon (Maercker and Zoellner, 2004; Zoellner and Maercker, 2006) whereby it may have “a *functional*, self-transcending or constructive side” as well as an “an *illusory*, self-deceptive, or dysfunctional side” (Zoellner and Maercker, 2006, pp 639–640, emphases in the original). Thus, while Hobfoll et al. (2007) acknowledge that “PTG following trauma may be what keeps people from sinking into apathy and surrender following trauma and it may be related to recovery and building of a more resilient self” (p. 362), they also assert that it is increasingly becoming clearer that PTG is not all positive.

In the current study, we set out to investigate the relation between veterans’ loneliness and their PTG. Loneliness has often been conceptualized as *perceived social isolation*, denoting the cognitive discrepancy between one’s desired and attained social connections (Cacioppo S. et al., 2015). Such discrepancy may relate either to the quality or quantity of relationships. As such, it has been argued that loneliness denotes the psychological pain that accompanies the perception that some of an individual’s relational needs are unfulfilled (Stein and Tuval-Mashiach, 2015b). To the best of our knowledge, the relation between the two phenomena has been the focus of only one study (Zeligman et al., 2017), wherein the researchers found that loneliness was not correlated with PTG, but nevertheless moderated the link between PTSD and PTG. Notwithstanding, the literature supports the postulation of four theoretically and empirically based scenarios, underscoring different dynamics relating the two phenomena.

The first hypothetical scenario suggests that high PTG may be a predictor of increased loneliness and is strongly related to the phenomenological properties of a veteran’s loneliness. Recent qualitative investigations (Stein and Tuval-Mashiach, 2015a; Stein, 2017a) suggest that the manifestation of veterans’ loneliness may be attributed primarily to their conviction that civilians cannot possibly comprehend the experiences of war and captivity, nor can they comprehend or identify with their post-traumatic aftermath (e.g., struggling with post-traumatic stress symptomatology). As these experiences are arguably incommensurable with quotidian life, it is argued that upon homecoming repatriated veterans and ex-POWs may sense that they are condemned to bear their torment alone. As veterans are inclined to retain their identities as warriors and eschew civilian identities, they may become ever more isolated and alienated within civilian society (e.g., Hoge, 2010; Smith and True, 2014; Ahern et al., 2015; Stein, 2017b).

This stratified phenomenon, termed *experiential loneliness* (Stein and Tuval-Mashiach, 2015a; Stein, 2017a,b), may also apply to PTG. As a person’s growth leads to a reevaluation of the Self, the world and others (Janoff-Bulman, 2006) this newly gained understanding may extensively differ from that of his or her surroundings. Thus, a veteran may become more isolated in the sense that the need for others to share their understanding and inner reality (Echterhoff et al., 2009), as well as the need to belong (Baumeister and Leary, 1995), remain unfulfilled and altogether ungratified. Furthermore, it is noteworthy that individuals who are characterized as self-enhancers (i.e., view themselves in an overly positive or unrealistic self-serving manner) may be viewed by others in a less favorable light (Paulhus, 1998). Thus, while being advantageous in the face of adversity, such characteristics may also be costly in the social context (e.g., Bonanno et al., 2005).

The second hypothetical scenario suggests that high PTG may be a predictor of decreased loneliness. As PTG includes an enhanced appreciation of relationships (Tedeschi and Calhoun, 1996, 2004), it stands to reason that those exhibiting such growth will feel more connected and hence less lonely. Indeed, as evident in a study among persons who have experienced loss (Stein et al., 2009), increases in personal growth may predict lower degrees of loneliness.

The third hypothetical scenario suggests that high loneliness may be a predictor of increased PTG. Loneliness has been viewed by existential thinkers as an inherent aspect of the human condition (e.g., Moustakas, 1961; Ettema et al., 2010; Mijuskovic, 2012). As such, it is argued that when people do not feel lonely it is because they manage to distract themselves from this existential predicament. Thus, as opposed to the evolutionary perspective, wherein loneliness is viewed as a signal that one must strengthen social ties (Cacioppo S. et al., 2015), from an existential perspective loneliness is viewed as a signal that one must reconnect internally with his or her Self to foster personal growth. In this respect, it is argued that the *state* of loneliness and the pain it entails as an *experience* differ from the *process* of loneliness that ultimately entails personal growth (Ettema et al., 2010). Some go as far as arguing that relinquishing the desire to connect with others on the experiential level is, in fact, a therapeutic goal. As combat veteran Daryl Paulson notes: “One of the first acts of courage in counseling is to acknowledge that one is essentially “alone” with one’s subjective experience... But, out of this aloneness, a client finds his or her real Self, a discovery that eventually provides a tremendous amount of satisfaction” (Paulson and Krippner, 2007, p. 114).

The fourth hypothetical scenario suggests that high loneliness may be a predictor of decreased PTG, whereas a sense of connectedness (i.e., low loneliness) may strengthen PTG. Being lonely may entail the endorsement of negative perceptions about one’s social desirability as well as negative evaluations of one’s social network (Cacioppo and Hawkley, 2009). Effective strategies devised to alleviate loneliness are aimed toward challenging such maladaptive cognitions (Masi et al., 2011). Given the aforementioned potentially illusory side of PTG (Zoellner and Maercker, 2006), as one feels more lonely following combat or captivity, one’s “bubble” of enhanced relational connection may burst. Consequently, a reevaluation of meanings may once again take place (Park, 2010; Larner and Blow, 2011) and gradually other positive perceptions may likewise diminish. In this respect, loneliness may be a catalyst for PTG disillusionment and a reduction in PTG may be expected as loneliness persists or exacerbates. As a complementary mirror image, it has been found that the social sharing of emotion may reduce loneliness and subsequently increase PTG (Rimé et al., 2010). As social sharing of emotion is related to greater emotional synchrony (Páez et al., 2015), the feeling of being “united in the same mind and in the same action” (p. 713) generates a sense of togetherness and therefore may strengthen rather than disillusion PTG.

In light of the above, in the current study we hypothesized that loneliness and PTG would be cross-sectionally (H1) as well as a prospectively related (H2). Nevertheless, we refrained from postulating the directionality of the relations between the two phenomena, both cross-sectionally and prospectively because, as apparent above, extant literature does not provide sufficient evidence to generate substantiated hypotheses. Therefore, concerning the directionality of effects we employed an exploratory rather than confirmatory approach, thus considering all four plausible scenarios delineated above and the tentative hypotheses that may be derived from them (H2.1–H2.4, respectively).

## Materials and Methods

### Participants and Procedure

The present study capitalized on data from a longitudinal study focusing on the psychosocial implications of war among combat veterans, some of whom were in captivity, from the 1973 Yom Kippur War (Solomon et al., in press). The overarching study consisted of four waves of measurement, 18, 30, 35, and 42 years after the war (1991, 2003, 2008, and 2015, respectively). However, since the PTG questionnaire was not administered in the 1991 measurement, the current study utilized only the data collected in the last three measurements. Participants completed the research questionnaires in the presence of a research assistant, either in their homes or in a location of their choice. All participants signed an informed consent agreement before taking part in each wave of the study. The Tel Aviv University Institutional Review Board has approved the ethical considerations of the study throughout the various waves of measurement.

To locate the veterans, we used Israel Defense Forces (IDF) records. According to Israel’s Ministry of Defense, 240 soldiers from the Israeli Army land forces were captured during the Yom Kippur War. In the 1991 assessment, 480 potential combat veterans were contacted, of whom 349 participated. Of these, 287 veterans participated in 2003 (T1; 51 could not be located or otherwise did not participate, 5 were deceased, and 6 could no longer participate due to mental deterioration). In 2008 (T2), the original 1991 veterans were re-contacted, and due to participant addition and attrition, 301 veterans participated (65 additional participants from the original sample, 71 could not be located or otherwise did not participate, 25 were deceased, 11 were living abroad, and 6 could no longer participate due to mental deterioration). In 2015 (T3), we once again contacted the original sample list of 1991, of whom 259 participated (22 could not be located or otherwise did not participate, 48 were deceased, 6 were living abroad, and 5 could no longer participate due to mental or physical deterioration).

The age of the veterans at T1 was: *M* = 53.5, *SD* = 4.6 (49–80 years); T2: *M =*56.8, *SD* = 5 (52–83 years); T3: *M* = 65.1, *SD* = 4.3 (59–80 years). Their level of education in years did not change significantly from T1 (*M* = 13.9, *SD* = 3.45) to T3 (*M* = 13.97, *SD* = 3.93) and ranged between 8 and 25 years. Forty-seven percent of the veterans were not working (retired) at T3. The amount of veterans that met criteria for PTSD at T1, T2, and T3 was: 82 (33%), 112 (50%), and 75 (35.3%) respectively.

### Data Analysis

Veterans were included in the sample only if they participated in at least two out of the three waves of measurement (*n* = 260). Overall, 9.9–28.9% of the data were missing across waves (28.9, 11.4, and 17.5% for PTG, and 26.6, 9.9, and 11.4% for loneliness, corresponding to T1, T2, and T3, respectively). Little’s missing completely at random (MCAR) test (Collins et al., 2001) was utilized in order to assess whether the missing values across waves were missing at random or missingness was otherwise biased. The analysis revealed that the data were missing completely at random [χ^2^(54) = 58.8, *p* = 0.30]. Hence, missing data were handled with maximum likelihood estimates (ML), which is widely endorsed in such circumstances (Schafer and Graham, 2002).

To examine the bidirectional relationship between PTG and loneliness over the three time-points, we employed an autoregressive cross-lagged modeling strategy (ARCL; Selig and Little, 2012) which allows for simultaneous assessment of whether earlier measures of PTG predict later measures of loneliness, and whether earlier measures of loneliness predict later measures of PTG, as well as cross-sectional relations between the two variables. In order to assess the appropriateness of the ARCL, we used the AMOS SEM software, version 23 (Arbuckle, 2014). We estimated the model’s fit by using the comparative fit index (CFI), Tucker-Lewis fit index (TLI), standard root-mean-square residual (SRMR), and the root-mean-square error of approximation (RMSEA). A model is judged as reasonably fitting the data when CFI, TLI, and 1-RMSEA are larger than 0.95, and SRMR is below 0.06 (Bollen and Curran, 2006). Missing data were handled with the case-wise maximum likelihood estimation for possible non-normality when running the model.

### Measures

#### Post-traumatic Growth

The Post-traumatic Growth Inventory (PTGI; Tedeschi and Calhoun, 1996) was used to measure PTG at three time points, listing 21-items anchored with regard to the Yom Kippur War. Participants were asked to indicate on a four-point scale the extent of change that occurred in their lives following the war (1 = *I didn’t experience this change at all*; 4 = *I experienced this change to a very great degree*). Based on this 21-item self-report scale, the total score was computed according to the five subscales: Relating to others, New Possibilities, Personal Strength, Spiritual Change, and Appreciation of Life. As noted above, the PTGI has demonstrated good reliability and concurrent validity as well as a replicable factor structure (e.g., Tedeschi and Calhoun, 1996; Kaler et al., 2011). In this study, the inventory showed excellent internal consistency regarding the total score for veterans throughout the various assessments (α = 0.93, 0.93, and 0.90 at T1, T2, and T3, respectively).

#### Loneliness

Though typically loneliness is measured using a comprehensive scale such as the UCLA loneliness scales (Russell et al., 1980), several studies have utilized as little as one general item indicating loneliness (e.g., Stessman et al., 2013). In the current study loneliness was measured as the average of two items from the 53-item Brief Symptom Inventory (Derogatis and Melisaratos, 1983). The two items were “*Feeling lonely”* and “*Feelings of loneliness even when you are around other people*.” For each item respondents were required to report the degree to which they were troubled by the problem depicted in the item in the past month, using a five-point Likert scale, ranging from 0 (*“not at all”*) to 4 (“*extremely*”).

## Results

**FIGURE 1:**
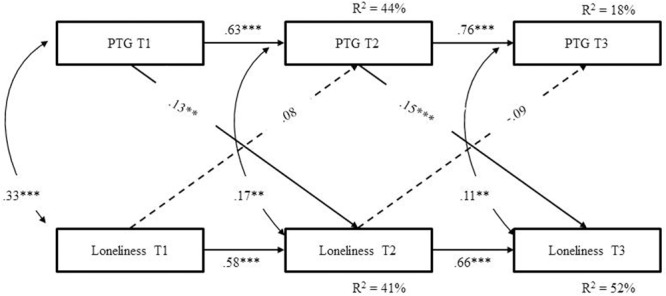
ARCL model for the associations between PTG and loneliness. PTG, post-traumatic growth; ^∗∗^*p* < 0.01, ^∗∗∗^*p* < 0.001.

**Figure 1** presents results from the ARCL assessment of the bidirectional relations between PTG and loneliness over time. The model yielded excellent fit to the data, χ^2^_(3)_ = 4.09, *p* = 0.25, CFI = 0.99, TLI = 0.99, 1-RMSEA = 0.96, RMSEA CI [0.97, 0.95], and SRMR = 0.01. The analysis revealed that the stability of PTG over time, as well as the stability of loneliness, was noticeably high and significant. Veterans with high levels of PTG or loneliness at T1 tended to have high levels of PTG or loneliness, respectively, at T2. Similarly, veterans with high levels of PTG or loneliness at T2 tended to have high levels of PTG or loneliness, respectively, at T3. All cross-sectional associations between PTG and loneliness were significant. The association between PTG at T2 and T3 residuals were likewise significant, indicating that there are additional factors that are not represented in this model that are associated with PTG. These residuals were controlled for in the current model. There was no association between the residuals of T2 and T3 loneliness factors, hence these were not controlled for in the model.

More importantly, the analysis revealed that the initial T1 level of PTG predicted the subsequent level of loneliness at T2, above and beyond loneliness stability, but not vice versa. Consistent with this cross association, we also found that the initial level of PTG at T2 predicted the subsequent level of loneliness at T3, above and beyond loneliness stability, but not vice versa. Thus, the model indicated that higher levels of PTG consistently predicted higher loneliness over time, while loneliness consistently failed to predict PTG.

## Discussion

In the current study we sought to shed light on the interplay between PTG and post-traumatic loneliness by prospectively investigating the links between these two phenomena among combat veterans across three waves of measurement 30, 35, and 42 years after the 1973 Yom Kippur War. As hypothesized, findings indicated that loneliness and PTG were consistently related cross-sectionally (H1) as well as prospectively (H2). Concerning the directionality of the relation, results revealed that the two phenomena were cross-sectionally positively correlated. Furthermore, longitudinal analyses revealed that while PTG consistently positively predicted subsequent degrees of loneliness, loneliness did not predict subsequent PTG levels. Thus, among the four potential scenarios considered at the outset, of those suggesting that PTG would be implicated in subsequent levels of loneliness, the first potentiality (H2.1) was supported by the findings while the second (H2.2) was rejected. Furthermore, both of the scenarios suggesting that loneliness may implicate PTG (H2.3–H2.4) were not supported by the findings. These findings suggest that as veterans evince higher degrees of personal growth in the aftermath of war or captivity, they are also likely to experience themselves as growing apart from their social connections, and thus become lonelier. In contrast, according to the findings, veterans who exhibited less PTG also perceived themselves as better socially connected, and thus reported lesser degrees of loneliness. Ostensibly, however, there exists no predictable pattern according to which degrees of loneliness predict PTG.

In explaining these findings it is imperative to elaborate upon the mechanisms of loneliness maintenance and the manner in which PTG may become a part of such processes. Loneliness is a product of an iterative process of evaluation and reevaluation that may result in its alleviation or maintenance and exacerbation. As noted above, the dominant notion in contemporary social science argues that loneliness represents a cognitive discrepancy between desired and perceived social connections (Perlman and Peplau, 1981; Cacioppo S. et al., 2015). This conceptualization implies constant reappraisals of and comparisons between expected and achieved connectedness (e.g., Russell et al., 2012). Furthermore, contemporary loneliness researchers, particularly in the field of social neuroscience, argue that humans, as social animals, are motivated to reconnect with others and that loneliness has developed via evolutionary processes to operate as an internal signal that such reconnection is to be sought (e.g., Cacioppo et al., 2014; Cacioppo J.T. et al., 2015; Cacioppo S. et al., 2015). In this respect, Qualter et al. (2015) argue that reconnection depends on the activation of the reaffiliation motive (RAM). However, the RAM may be hampered by negative cognitions. Evaluating one’s social connections while feeling socially isolated (i.e., lonely) may lead to attentional and memory biases as well as behavioral confirmation processes through which lonely individuals are increasingly vigilant regarding negative social clues. Thus, they engage in more negative social interactions and elicit evidence that ostensibly confirms that they have little personal or social value (Cacioppo and Hawkley, 2009; Cacioppo J.T. et al., 2015). Therefore, loneliness eliciting perceptions (e.g., “*I am inherently different from those surrounding me*”) that are not refuted or otherwise rejected by the individual, even if they are seemingly positive in their own right (e.g., “*I am inherently better than others*”), may result in the maintenance or exacerbation of loneliness rather than facilitate its alleviation as time progresses. Acknowledging the qualitative properties of the veteran’s loneliness, this process may account for our finding that high levels of PTG predicted high levels of loneliness.

As noted above, qualitative observations suggest that veterans’ loneliness is first and foremost a sense of *experiential loneliness* (Stein and Tuval-Mashiach, 2015a; Stein, 2017a,b) – the persuasion that one is isolated because no one among his or her close relations shares the experiences he or she has undergone during and after the war. Acknowledging that loneliness invariably consists of a sensation that certain relational provisions are not fulfilled (Stein and Tuval-Mashiach, 2015b), it is stressed that veterans’ experiential loneliness entails several ungratified relational needs (Stein, 2017b) that are potentially related also to PTG. Below we address three such needs more explicitly to elucidate the manner in which the various aspects of this loneliness may relate to PTG.

Several lines of research in social psychology indicate that people have a profound need to sense commonality with others’ inner states and world views (e.g., Pinel et al., 2006; Echterhoff et al., 2009). As veterans’ traumatic backgrounds give rise to a positive outlook on life and as they come to perceive themselves and others in a more favorable light (Janoff-Bulman, 2006), they may also long to be surrounded by others who share these views. Comparing their perspectives with those in their surroundings and realizing that others do not share their subjective views of reality, it may be that the need for a shared inner state is compromised, and loneliness materializes.

Belongingness is yet another fundamental human need (Baumeister and Leary, 1995), and is germane to the formation of one’s identity (e.g., Hagerty et al., 1992) as well as to the manifestation of loneliness (Baumeister and Leary, 1995). Veterans’ view of themselves and others, whether positive or negative, is strongly grounded in their military experiences, and as such define who they are *as veterans* (Hoge, 2010). PTG adds a positive stratum to the trauma survivor’s identity (Maitlis, 2009) and thereby represents an integral part of post-traumatic narrative reconstruction and meaning-making processes (e.g., Pals and McAdams, 2004; Park, 2010). That is to say that PTG is the process whereby one reconstructs his or her “Self” in the aftermath of trauma along the lines of personal growth (Pals and McAdams, 2004). However, such growth may further sever the individual from his or her social surroundings because, from the individual’s perspective, it renders him or her different from such surroundings. In this respect, a civilian-military gap is increasingly realized, wherein the “veteran identity” is contrasted by veterans to the “civilian identity” primarily in that it encapsulates and engenders different world-views. This may foster contempt toward those who do not share this view (i.e., toward civilians; Rahbek-Clemmensen et al., 2012). Concomitantly, observations dating as far back as the World Wars (Waller, 1944; Schuetz, 1945) and through to the aftermath of contemporary military conflicts (e.g., Brewin et al., 2011; Smith and True, 2014; Ahern et al., 2015; Stein and Tuval-Mashiach, 2015a; Orazem et al., 2016) poignantly demonstrate that veterans throughout the decades have felt alienated in civilian society. This is attributed to returning veterans’ realization that their participation in the wars has rendered them altogether different people than they used to be prior to enlistment and deployment. Consequently, some feel that they no longer belong among civilians who remain oblivious to the military culture and to the stressors of war and their far-reaching implications. Acknowledging that veterans compare their views to those of civilians it may be recognized that endorsing PTG perceptions emphasizes social differences rather than similarities. Therefore, PTG may contribute to the formation of an identity that cultivates and fortifies veterans’ sense of “*experiential alienation*” (Stein, 2017b, p. 17) – the sense that one does not belong due to who he or she is. When a person’s sense of belongingness is thwarted, this may foster more loneliness.

Finally, a major component in fostering a sense of social connection is the notion that one may count on his or her social network for social support (Rook, 1984; Vaux, 1988a,b). It has been argued that veterans’ loneliness entails a discrepancy between negative internal sensations and a positive façade that is outwardly expressed, often at the expense of utilizing necessary social support (Stein, 2017a,b). Individuals who endorse PTG generate narratives that portray and promulgate a positively altered identity (Pals and McAdams, 2004). However, as many studies indicate (e.g., Pietrzak et al., 2010; Shakespeare-Finch and Lurie-Beck, 2014; Tsai et al., 2015), PTG does not imply symptomatic resilience and often coexists with and is complemented by PTSD symptomatology. Veterans who boast PTG may misleadingly convey that they have been resilient to the trauma, when in actuality they face the trauma’s negative ramifications alongside the positive. Attempting to maintain a sense of consistency, they may refrain from revealing their hardship, and thus choose to cope alone with their post-traumatic emotional turmoil and abstain from soliciting needed support. Ultimately, coping alone may foster a sense of being terribly lonely. Undeniably, while the explanations suggested above may fit the findings in the current study, they are nevertheless speculative. Future research is needed in order to unravel the nature of the relations between loneliness and PTG.

### PTG – Simultaneously Adaptive and Maladaptive

A major “allegation” concerning PTG’s negative implications has been that PTG is not veridical in that it represents illusory perceptions rather than authentic growth (e.g., Zoellner and Maercker, 2006; Hobfoll et al., 2007; Lahav et al., 2016). However, it is our view that this conclusion is not warranted and is indeed not a necessary interpretation of the results in studies promoting this view, and nor is it the only logical conclusion of the present investigation. Rather, following the long-standing philosophical principle that opposites coexist (McGill and Parry, 1948), we favor what may be termed a “Zebra Philosophy” view. Accordingly, phenomena simultaneously entail both positive and negative aspects, black and white rather than shades of gray, as in the Zebra’s stripes. The goal of intervention throughout various domains is, therefore, to identify both positive and negative aspects for what they are and strive to minimize the negative and preserve or enhance the positive. From a zebra philosophy perspective, PTG may represent authentic gains and real positive alterations in world views and *at the same time* foster and coexist alongside negative outcomes. In this respect, much like the trauma itself may result in PTSD and PTG simultaneously, PTG may be adaptive in some respects and maladaptive in others, concurrently.

The adaptive or maladaptive nature of PTG is to be judged by its derivatives, and as such needs refinement. As Hobfoll et al. (2007, p. 359) stress, “post-traumatic growth may be a marker of positive adaptation when accompanied by actions, not solely cognitive maneuvers.” Regarding loneliness, this amounts to the need to identify those aspects of PTG that promote social withdrawal and foster social disconnection and strive to distinguish them from those that facilitate enhanced well-being and better functioning. The goal must be to relinquish the former while maintaining the latter. Along these lines, Westphal and Bonanno (2007, p. 422) assert that, “pragmatic coping strategies, such as self-enhancing biases, are often associated with longer-term social liabilities. . . . Dismissing self-enhancers as dysfunctional, however, obscures the coping advantage these individuals appear to hold when they are confronted with events that present a significant threat to the self.”

For instance, an Israeli combat veteran who told his life-story in the testimonial project that has been the focus of the initial qualitative investigation of veterans’ loneliness (Stein and Tuval-Mashiach, 2015a) has noted the following:

She [his wife] would ask for minor things. This is perhaps an insight of someone who understands how life can vanish in a second. That... there are priorities in life. So what? I said this you said that, and the other thing – nonsense. At the end of the day, in a second a person can vanish. So let’s see what are the essential things in life. And whoever was not in this situation [of the war – J.Y.S] does not understand at all what it is that we are talking about, just does not understand what we’re talking about. And the truth is that here there were many gaps, because I… by me priorities in life have totally changed and by her still... The little things that may have perhaps created a more pleasant atmosphere, but for me are not the essence, are not the most important... not... it won’t change the world... and about this we had many arguments.

This excerpt demonstrates the aforementioned experiential loneliness, and the pathway through which growth may hamper veterans’ marital relationships. Indeed, the notion that PTG may result in greater friction within close relationships is supported by recent findings indicating that PTG may be associated with decrements in dyadic adjustment among this cohort of veterans and their wives (Lahav et al., 2017). However, this does not necessarily indicate that PTG is illusory or all bad in such an instance. Rather, PTG may have salubrious implications for the individual and at the same time sabotage his or her close relationships. Interventions in such cases should identify both implications and, while commending and encouraging personal growth, also explore alternative actions that may delay conflict and promote adaptive communication, mutual understanding, and perhaps also mutual growth.

Another example may be given from the social domain rather than the romantic, demonstrating two phases of social detachment: experience driven differentiation and social intolerance. An ex-POW from the Stein and Tuval-Mashiach (2015a) study noted that his enhanced enjoyment of nature after repatriation was what essentially differentiated him from people who had never experienced such adversity. In his words:

I am sure of it, that my enjoyment of things that people ordinarily don’t pay attention to and are not aware of their existence, my enjoyment of these things is simply immense. For instance, if I hear the sound of the wind in the leaves on the trees... If I hear the sound of flowing water of waves, the flow of water in streams, see green nature, I experience that, I think that I experience that in a way that other people don’t. To hear the sound of birds tweet, I, it drives me insane, in positive enjoyment. Such little things that people don’t pay attention to in their everyday.

However, it is not merely that his personal growth has rendered him different from others, but ultimately that it has altered his evaluation of potential companions. Indeed, in a later section of his life-story he noted that his enhanced appreciation of life made him intolerant to others’ preoccupation with what he considered unimportant and petty:

I can’t waste my time on relationships, whether at home or with friends that are not of a certain caliber. I can’t listen to people speaking about shopping, about possessiveness and “where we went” and “what we bought.” I can’t. It drives me out of my mind, out of my mind! I don’t keep in my company people of this kind. I can’t.

As in the former example, PTG has limited this veteran’s capacity to tolerate social relationships that do not demonstrate growth. In this respect, both excerpts echo Jaime Primak Sullivan’s quip in the epigraph. Furthermore, as a complementary social note, it is noteworthy that previous findings show that while self-enhancing biases may indeed be advantageous for the individual, others may nevertheless perceive such individuals in a less favorable light (e.g., Paulhus, 1998; Bonanno et al., 2005), which may increase their sense of thwarted belongingness. However, this does not imply that veterans must relinquish or forfeit their growth. Rather, to increase social integration and decrease the likelihood that loneliness and withdrawal transpire veterans may be encouraged to examine the maladaptive social cognitions that they have derived from their growth, and alter them if necessary.

### Limitations

The findings in the current study bear several noteworthy limitations. First, findings may be affected by our choice of measurement tools. The PTGI (Tedeschi and Calhoun, 1996) is but one of several optional tools for measuring the positive outcomes of adversity and different measures may yield different results (Joseph and Linley, 2008b). Moreover, the assessment of loneliness in the present investigation was done using two items from the BSI (Derogatis and Melisaratos, 1983), rather than using a more comprehensive standardized measure of loneliness such as the commonly used UCLA loneliness scale (Russell et al., 1980). As scales of both PTG (Joseph and Linley, 2008b) and loneliness (Hawkley et al., 2005) may measure different types or aspects of growth and loneliness respectively, using such tools may shed further light on the question at hand. Furthermore, it has been argued that that asking about loneliness explicitly may result in under-reporting, especially among men (e.g., Marangoni and Ickes, 1989) and this recommendation is typically incorporated in more comprehensive loneliness questionnaires. Future studies should, therefore, replicate the current findings with diverse and comprehensive assessment tools.

Secondly, the current study design cannot determine causality, although it strongly suggests it. This is due to three reasons. First, the study does not include baselines for any of the study variables, nor do we have data preceding the trauma. Secondly, one cannot negate the possibility that loneliness was not the outcome of PTG but rather affected by other factors that are associated with PTG. Finally, even assuming that PTG does indeed impact loneliness, as suggested above, several intervening factors may be at play. Future investigations should, therefore, replicate and expand the current findings by including baseline, pre-trauma and post-trauma measures of loneliness and PTG, as well as additional variables that may mediate or moderate the effect. Such a longitudinal investigation would ideally necessitate considerably larger samples, and would do well to investigate also the aftermath of traumas other than war.

Notwithstanding these limitations, in revealing the possible negative effects that PTG may have for perceived social isolation (i.e., loneliness), the current study bears clinical and scientific implications. From a clinical perspective, the findings suggest that when patients report PTG perceptions, it is important to probe for associated perceptions of social isolation. In cases wherein PTG indeed leads to loneliness, it may be useful to further probe whether the maintenance of such perceptions impedes the veteran’s reintegration efforts. This is in light of findings that imply that social barriers may hinder veterans’ reintegration into civilian society (Sayer et al., 2010), and thwarted belongingness may be related to veteran suicidality (e.g., Anestis et al., 2009). Furthermore, loneliness and lack of social support have been identified as critical threats to health and mortality (e.g., Holt-Lunstad et al., 2010, 2015, 2017) and hence should be targeted in clinical interventions (Masi et al., 2011). Clinicians should assess what aspects of PTG serve as a resource and recovery capital and what aspects are associated with greater loneliness, and for whom. As they do so, they may provide psychoeducation as to the potential processes linking PTG and loneliness and foster more socially-adaptive reactions to personal growth. From a scientific perspective, it may be useful to seek empirically based discernments between PTG-induced loneliness and traumatization-induced loneliness, both phenomenologically (i.e., the intricacies of the lived experience) and clinically (i.e., pathological ramifications). Both qualitative and quantitative investigations may be extremely informative in such an endeavor.

## Author Contributions

JS conceptualized the study, wrote up the manuscript’s ‘Introduction’ and ‘Discussion’ sections, and edited the ‘Materials and Methods’ and ‘Results’ sections. YL conducted the analysis and wrote up the initial draft for the ‘Materials and Methods’ and ‘Results’ sections. RB contributed both in editing the entire paper and in making literature recommendations. ZS was head of the research lab, and supervised the collection of the data for the study and the study as a whole. She also made recommendations throughout the work.

## Conflict of Interest Statement

The authors declare that the research was conducted in the absence of any commercial or financial relationships that could be construed as a potential conflict of interest. The reviewer HG and handling Editor declared their shared affiliation.
